# Role of Virulence Factors of Trypanosomatids in the Insect Vector and Putative Genetic Events Involved in Surface Protein Diversity

**DOI:** 10.3389/fcimb.2022.807172

**Published:** 2022-04-28

**Authors:** Artur Leonel de Castro Neto, José Franco da Silveira, Renato Arruda Mortara

**Affiliations:** Departamento de Microbiologia, Imunologia e Parasitologia, Escola Paulista de Medicina, Universidade Federal de São Paulo, São Paulo, Brazil

**Keywords:** Trypanosomatids, virulence factors, insect vectors, host parasite interaction, parasite genetic variability

## Abstract

Trypanosomatids are flagellate protozoans that can infect several invertebrate and vertebrate hosts, including insects and humans. The three most studied species are the human pathogens *Trypanosoma brucei*, *Trypanosoma cruzi* and *Leishmania* spp. which are the causative agents of Human African Trypanosomiasis (HAT), Chagas disease and different clinical forms of leishmaniasis, respectively. These parasites possess complex dixenous life cycles, with zoonotic and anthroponotic stages, and are transmitted by hematophagous insects. To colonize this myriad of hosts, they developed mechanisms, mediated by virulence factors, to infect, propagate and survive in different environments. In insects, surface proteins play roles in parasite attachment and survival in the insect gut, whilst in the mammalian host, the parasites have a whole group of proteins and mechanisms that aid them invading the host cells and evading its immune system components. Many studies have been done on the impact of these molecules in the vertebrate host, however it is also essential to notice the importance of these virulence factors in the insect vector during the parasite life cycle. When inside the insect, the parasites, like in humans, also need to survive defense mechanisms components that can inhibit parasite colonization or survival, e.g., midgut peritrophic membrane barrier, digestive enzymes, evasion of excretion alongside the digested blood meal, anatomic structures and physiological mechanisms of the anterior gut. This protection inside the insect is often implemented by the same group of virulence factors that perform roles of immune evasion in the mammalian host with just a few exceptions, in which a specific protein is expressed specifically for the insect vector form of the parasite. This review aims to discuss the roles of the virulence molecules in the insect vectors, showing the differences and similarities of modes of action of the same group of molecules in insect and humans, exclusive insect molecules and discuss possible genetic events that may have generated this protein diversity.

## Introduction

The three most studied species of trypanosomatids are the human pathogens *Trypanosoma brucei*, *Trypanosoma cruzi* and *Leishmania* spp. which are the causative agents of Human African Trypanosomiasis (HAT), Chagas disease and different clinical forms of leishmaniasis, respectively. These parasites are flagellate protozoans that possess complex dixenous life cycles, infecting several invertebrate and vertebrate hosts, and are transmitted by hematophagous insects (Kaufer et al., 2017; Sylvia and Aldo, 2019). The transmission from the mammal host to the insect vector occurs when tsetse flies, triatomine bugs or sand flies ingest a blood meal, taking parasites, which may be inside infected cells or free-living in the bloodstream. Following ingestion, the parasites differentiate into their insect form and colonize several areas of the vector digestive system. To complete the life cycle, these parasites are transmitted back to the vertebrate hosts *via* different mechanisms: *T. brucei* is inoculated in the host by tsetse flies, while *T. cruzi* is eliminated with feces or urine by triatomine bugs and *Leishmania* is regurgitated by sand flies (Cholewiński et al., 2015; Geiger et al., 2016).

To persist in this variety of hosts, they developed mechanisms, mediated by their virulence factors, to infect, propagate and survive in these different environments. Many studies have reported the impact of these molecules in the vertebrate host. They are mostly composed of parasites’ surface proteins that perform roles of evading the immune system by inhibition of cytokines production, protection against complement-mediated lysis, adhesion, invasion and survival inside host cells (Geiger et al., 2016; Stijlemans et al., 2016; de Castro Neto et al., 2021). However, it is also essential to discuss the importance of the virulence factors in the insect vector during the parasite life cycle. In a similar way to the human infection, once the parasites are ingested by insects, they need to endure defense mechanisms and structures that are in place to inhibit their colonization and survival. The anatomy and physiology of the anterior gut, the midgut peritrophic membrane barrier, digestive enzymes activity and excretion alongside the digested blood meal are a few examples of these mechanisms (Franco et al., 2012).

The parasite’s protection in this situation is often performed by the same group of virulence factors used in the mammalian host with just a few exceptions, in which a certain protein is expressed specifically by the insect vector form of the parasite (Schlein et al., 1990; Aksoy, 2019; Herreros-Cabello et al., 2020). The presence of these molecules with function in both, vertebrate and invertebrate hosts, may be a result of trypanosomatid evolution, since it has been proposed that they may have been first originated as an insect-borne parasite, which later became digenetic (Hamilton et al., 2004).

This review aims to display the role of these virulence factors in the insect vector, showing how the same group of molecules have different modes of action in insect, when compared to their roles during the human infection, and discuss possible genetic events that may have generated host-specific protein subsets.

## Virulence Factors in Insects

### T. brucei

During the invertebrate host life cycle, once ingested by the insect, *T. brucei* differentiates from bloodstream to procyclic forms. The onset of the differentiation process may be associated with the parasite exposure to the insect’s proteases and the higher pH in the tsetse fly midgut (Ponte-Sucre, 2016). At this point of the life cycle, the parasite’s membrane is still covered by the variant surface glycoprotein (VSG), anchored by glycosylphosphatidylinositol (GPI), that is the most abundant surface protein on bloodstream forms, responsible for evading the host’s adaptative immune response (Horn, 2014; Pays et al., 2014). Following parasite ingestion by the insect, a change in the trypanosome surface coat occurs and the VSGs are quickly removed by proteolysis and GPI hydrolysis, catalyzed by major surface proteases (MSP) and phospholipase C (PLC), respectively (Moreno et al., 2019) and released into the midgut of the tsetse fly (Ponte-Sucre, 2016). Once in the midgut, cells responsible for producing the peritrophic matrix (PM), a semipermeable, chitinous barrier that lines the midgut, incorporate the released VSGs. The incorporation leads to a decrease in expression of the tsetse fly microRNA (mir-275), interferes with the Wnt-signaling pathway and the activity of Iroquois/IRX transcription factor family (Aksoy et al., 2016). These events impair the role of the PM that functions as a barrier to infection by pathogens, and promote parasite proliferation in the gut during the early stages of the infection process (Aksoy et al., 2016; Ponte-Sucre, 2016; Aksoy, 2019).

At the same time, while in the insect midgut, the VSGs of trypanosome surface coat are replaced by procyclins, a stage-specific group of proteins which is the main acceptor of sialic acid in *T. brucei* (Engstler et al., 1993; Pontes de Carvalho et al., 1993; Montagna et al., 2002; Jackson, 2015). They comprise a small multigene family, anchored by GPI that is differentially expressed during infection (Urwyler et al., 2005). Procyclic trypomastigotes use a GPI-anchored trans-sialidase to transfer sialic acids from the host glycoconjugates to the procyclins and glycosylphosphatidylinositols (GPIs) expressed on their surface (Engstler et al., 1993; Pontes de Carvalho et al., 1993; Montagna et al., 2002; Nagamune et al., 2004). Surface sialic acids protect the trypanosomes from the hostile environment of the midgut of tsetse flies (Nagamune et al., 2004). Procyclins may also be involved in the protection of the parasite surface against the activities from digestive enzymes mediated by their C-terminus proteases resistance domain (Acosta-Serrano et al., 2001b), hydrolases in the tsetse fly midgut and could be essential for the differentiation of procyclic trypomastigote to the metacyclic form (McConville et al., 2002).

### T. cruzi

Relative to *T. cruzi*, by the time these parasites enter the gut of the insect vector through the blood meal, they are challenged by the invertebrate host defense mechanisms and components aimed to digest the ingested blood. These components comprise hemolytic factors, proteases, oxygen and nitrogen radicals and components of the vector humoral immune system (Garcia et al., 2007). To assist the parasite survival during the life cycle in the insect, *T. cruzi* Type-1 glycoinositolphospholipids (GIPLs) molecules (Colli and Alves, 1999), similar to the GIPLs) present in *Leishmania* and widely present on the surface of *T. cruzi* epimastigotes, are responsible for parasite attachment on the insect’s gut (Garcia et al., 2007; Nogueira et al., 2007).

Mucins are also a group of proteins with great importance during the establishment of infection. In vertebrates, they are responsible for cell adhesion, immunomodulation of host defense and complement system evasion (Almeida et al., 1999; Colli and Alves, 1999; Acosta-Serrano et al., 2001a). In the insect vector, they are, perhaps, one of the best characterized group of proteins (TcSMUG) involved in the protection of *T. cruzi* expressed during this stage of the parasite life cycle. TcSMUG are present in the epimastigote form and are categorized in two groups: L (large) and S (small), based on their mRNA size and structure (Di Noia et al., 2000). The TcSMUG S found in the epimastigote and metacyclic trypomastigote stages are major acceptors of sialic acid transferred by trans-sialidases (Schenkman et al., 1993; Pech-Canul et al., 2017) and were identified as the backbone for the GP35/50 mucins (Herreros-Cabello et al., 2020). They were shown to be resistant to proteases, which may protect the parasite in the insect vector intestinal tract (Mortara et al., 1992). On the other hand, the TcSMUG L, exclusive to epimastigotes, are glycoconjugates that do not accept sialic acids (Urban et al., 2011), and might be implicated in adhesion to the vector midgut surface (Gonzalez et al., 2013).

In *T. cruzi*, the glycan structures on these mucins may vary depending on the host. They have also shown to possess stage-specific variations, observed throughout the parasite life cycle, with strain specific-modifications present in the oligosaccharides branching from the peptide backbone (Previato et al., 2004; Mendonça-Previato et al., 2005; Giorgi and de Lederkremer, 2020). The role of these glycan structures has also been studied in GIPLs and Gp35/50 kDa mucins relative to parasite attachment to the insect digestive tract *via* assays using native molecules purified from the epimastigotes, recombinant Gp35/50 kDa mucin-like proteins, transgenic epimastigotes over-expressing Gp35/50 kDa mucins and chemically synthesized oligosaccharides (Mendoza et al., 2006; Nogueira et al., 2007; Cámara et al., 2019; Giorgi and de Lederkremer, 2020).

Adhesion of epimatigotes to the insect midgut was inhibited by GIPLs purified from epimastigotes of Y strain (*T. cruzi* lineage TcII), while mucins had no such inhibitory effect on the parasite binding to the midgut (Nogueira et al., 2007). On the other hand, several lines of evidence indicated that Gp35/50 kDa mucins play a role in the parasite attachment towards triatomine hindgut rather than midgut tissues (Cámara et al., 2019; Giorgi and de Lederkremer, 2020). Namely, a) native and recombinant Gp35/50 kDa mucins bind to the *Triatoma infestans* rectal ampoules*;* b) ex *in vivo* attachment assays showed that the binding of transgenic epimastigotes over-expressing Gp35/50 kDa mucins to the insect hindgut was 2-3 fold higher than those of wild type epimastigotes; c) synthetic oligosacharides based on Gp35/50 kDa mucins specifically inhibited the epimastigote attachment to *T*. *infestans* rectal ampoule.

The 63 kDa glycoprotein (gp63), another widely studied *Leishmania* virulence factor, encoded by a multigene family highly expanded in *T. cruzi* genome (El-Sayed, 2005), may contribute to the parasite’s virulence in the insect stage *via* its metalloprotease activity (Cuevas et al., 2003). A study has also shown the participation of gp63 in the parasite’s adhesion to *Rhodnius prolixus* midgut *via* assays using divalent metal chelators or anti-Tcgp63-I antibodies caused an impaired *T. cruzi* adhesion to the vector midgut (Rebello et al., 2019).

As for others specific *T. cruzi* virulence factors, cruzipains, which are cysteine proteases responsible for cell invasion and immune evasion during the vertebrate host infection (McKerrow et al., 2006), were also reported to contribute to parasite adhesion to the insect’s midgut (Uehara et al., 2012). Trans-sialidases (TS) comprise a multigene family that plays an important role by transferring sialic acid to mucin-like proteins on the cell surface and facilitate invasion in the vertebrate host (Schenkman et al., 1994). TS family also has a subset of genes expressed exclusively on epimastigotes (Schenkman et al., 1994; Briones et al., 1995; Retana Moreira et al., 2021) and they seem to possess roles of protection from glycolytic enzymes and adhesion to the insect’s gut (Schenkman et al., 1994).

### *Leishmania* spp.

Upon insect ingestion, *Leishmania* spp., tend to use the same classes of molecules that also help the parasite to evade the human’s immune system, with their roles summarized on **Table 1**. Generally during the vertebrate host life cycle, glycoconjugates like lipophosphoglycans (LPG), proteophosphoglycans (PPG) and gp63 perform roles of attachment to the host’s cells, modulation, and evasion of the immune system components (de Castro Neto et al., 2021). To survive in the insect, following blood meal ingestion, these parasites, still in the intracellular amastigote stage, start their differentiation to promastigotes, exit the macrophages and are exposed to midgut hydrolytic enzymes. However, a dense glycocalyx barrier formed by LPG and PPG provides protection against the action of these enzymes and also inhibit the release of midgut proteases (Schlein et al., 1990). LPG also plays a significant role by mediating promastigotes adhesion to midgut epithelial cells, which prevents the parasite elimination with the whole blood meal and ensures the completion of its life cycle (Sacks et al., 2000; Ilg, 2001).

**Table 1:** Groups of virulence factors from *T. brucei*, *T. cruzi* and *Leishmania spp*., with different functions on vertebrate and invertebrate hosts.

Virulence factor	Host	Function	Reference
***T. brucei***			
Variant surface glycoprotein (VSG)	Vertebrate	Adaptive immune response evasion	(Horn, 2014; Pays et al., 2014)
	Insect	Reduce the peritrophic matrix barrier function and help the parasite establish infection of the gut	(Aksoy et al., 2016)
Procyclin	Vertebrate	Not present	
	Insect	Protection of the parasite surface against digestive enzymes and metacyclicogenesis	(Acosta-Serrano et al., 2001b; McConville et al., 2002)
***T. cruzi***			
Glycoinositolphospholipids (GIPLs)	Vertebrate	Downregulation of immunomodulatory components on macrophages and dendritic cells	(Brodskyn et al., 2002)
	Insect	Adhesion to the insect’s gut	(Garcia et al., 2007; Nogueira et al., 2007)
Glycoprotein gp63	Vertebrate	No clear function detected on vertebrate host cell stages (trypomastigote and amastigote)	(Cuevas et al., 2003)
	Insect	Metalloprotease activity in the insect stage	(Cuevas et al., 2003)
		Adhesion to the insect’s gut	(Rebello et al., 2019)
Cruzipain	Vertebrate	Cell invasion and immune evasion	(McKerrow et al., 2006)
	Insect	Adhesion to the insect’s gut	(Uehara et al., 2012)
Trans-sialidases (TS)	Vertebrate	Transfer of sialic acid residues	(Schenkman et al., 1994)
		Host cell adhesion and evasion	
	Insect	Protection from the gut glycolytic enzymes	(Schenkman et al., 1994)
		Adhesion to the insect’s gut	
Mucins	Vertebrate	Cell adhesion	(Almeida et al., 1999; Acosta-Serrano et al., 2001a)
		Immunomodulation of host defense	
		Complement system evasion	
	Insect	TcSMUG S	(Mortara et al., 1992; Herreros-Cabello et al., 2020)
		Protection against proteases in the insect’s intestines	
		TcSMUG L Adhesion to the insect’s gut	(Gonzalez et al., 2013)
***Leishmania spp.***			
Lipophosphoglycans (LPG)	Vertebrate	Complement system evasion	(Forestier et al., 2015)
		Adhesion to macrophages	(Cunningham, 2002)
		Impairment of phagosome maturation and acidification	(Lodge et al., 2006; Borges et al., 2021)
	Insect	Protection from insect’s digestive enzymes	(Schlein et al., 1990)
		Adhesion to the insect’s gut	(Sacks et al., 2000; Ilg, 2001)
Proteophosphoglycans (PPG)	Vertebrate	Adhesion to macrophages	(Piani et al., 1999)
		Modulation of macrophages at the early stage of infection	
	Insect	Protection from insect’s digestive enzymes	(Schlein et al., 1990)
		Involved with the production of the Promastigote Secretory Gel (PSG), that may be responsible for the regurgitation of the parasites by the sand flies during the blood feeding	(Franco et al., 2012; Giraud et al., 2019)
Glycoprotein gp63	Vertebrate	Complement system evasion	(Isnard et al., 2012; Shao et al., 2019)
		Macrophage binding and entry	(Olivier et al., 2012)
		Downregulation of cellular compounds aimed to parasite elimination	(Podinovskaia and Descoteaux, 2015)
	Insect	Adhesion to the insect’s gut	(D’Avila-Levy et al., 2006a; Pereira et al., 2009; Soares et al., 2017)
		Parasite protection against the insect’s defense mechanisms	(D’Avila-Levy et al., 2014)

Besides the PPGs actions in interacting and modulating the mammalian immune system (Piani et al., 1999), it seems that this group of proteins main role is the maintenance of the digenetic life cycle, especially during the transmission process from the vector to the vertebrate host (Rogers et al., 2004). To that end, *Leishmania ssp.* produces a gel-like obstruction in the sand fly anterior midgut, called Promastigote Secretory Gel (PSG), composed mainly by secretion of filamentous proteophosphoglycan (FPPG) and other related molecules. This obstruction may stimulate to the regurgitation of the parasites by the sand flies during the blood feeding (Franco et al., 2012; Giraud et al., 2019). The PSG was also reported to be released with the *Leishmania* during transmission and showed to positively influence the development of the disease (Rogers et al., 2004). Furthermore, PSG extracted from the insect vector, produced by *L. major* and *L. tropica*, was able to intensify infections in mice (Giraud et al., 2019).

Gp63, considered one of the main surface proteins in *Leishmania*, has been widely studied specially for its immune evasion roles during the vertebrate host infection, briefly summarized on **Table 1**. Furthermore, this group of proteins also play significant roles in the insect vector, mainly associated with parasite adhesion to the insect’s intestinal epithelium and degradation of its protein components (D’Avila-Levy et al., 2006a; Pereira et al., 2009). This degradation may be involved with the parasite’s actions against the insect’s defense molecules and may have significant roles in the parasite’s nutrition pathway, due to its function as an endopeptidase, broad substrates specificity and optimum pH (D’Avila-Levy et al., 2014). Despite it has been hard to obtain direct evidence of the protein’s nutritional role in insects, the adhesion events have been widely demonstrated both in monoxenous trypanosomatids, e.g., *Crithidia guilhermei* (D’Avila-Levy et al., 2006a), *Leptomonas* spp. (Pereira et al., 2009), *Angomonas deanei* (D’Avila-Levy et al., 2008) and *Herpetomonas muscarum* (Nogueira de Melo et al., 2006); and dixenous trypanosomatids, e.g., *Phytomonas serpens* (D’Avila-Levy et al., 2006b), *T. cruzi* (Rebello et al., 2019) and *Leishmania* spp. (Soares et al., 2017). In Leishmania, *in vitro* and *in vivo* studies in different species showed parasites that had this protein blocked by anti-gp63 antibodies, chemical chelators (Soares et al., 2017) or when down-regulated (Jecna et al., 2013) substantially inhibited attachment in the midgut of insects from the *Lutzomyia* genus. A similar down-regulation study also showed that gp63 seemed to be important for *L. amazonensis* early stage development in *Lutzomyia longipalpis* (Hajmová et al., 2004), while a complete gene deletion in *L. major* showed the opposite result in the sand fly vector (Joshi et al., 2002). This may show that gp63 may present divergent roles in different *Leishmania* species relative to interactions with their respective insect vector. These events may also be a consequence of gp63 divergent gene copy numbers and sequence variability across the *Leishmania* species, which will be further discussed in this review, that may allow the parasite to infect a variety of insect hosts. Another hypothesis that should also be considered is the redundant roles of the other cited virulence factors in insects. In case of total absence or decreased amount of gp63, it would be compensated by other molecules like LPG and PPG that perform similar adhesion and protection roles, as displayed in **Table 1**.

**Figure 1:**
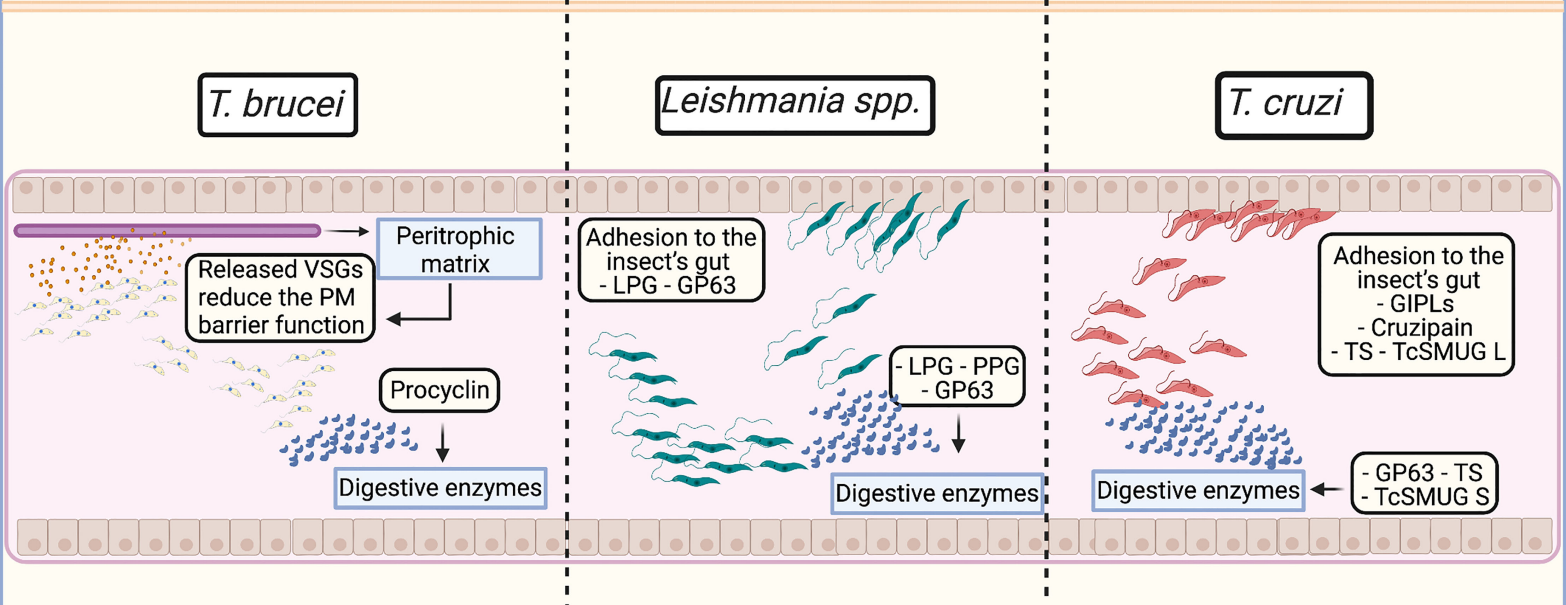
Virulence factors roles in their insect vectors intestine tract. ***T. brucei*:** Variant surface glycoprotein (VSGs) released from the membrane are incorporated by the peritrophic matrix, reducing its protective function by interfering with the host cell internal pathways. Following VSG release, the surface coat is replaced by procyclins that may protect the parasite against digestive enzymes and hydrolases in the tsetse fly midgut. ***Leishmania* spp.:** Inside the invertebrate vector intestines, lipophosphoglycans (LPGs) and gp63 ensure the parasite adhesion to the midgut epithelial cells and alongside proteophosphoglycans (PPGs), protect the parasite against the action of host digestive enzymes. ***T. cruzi*:** The parasite adhesion is performed by glycoinositolphospholipids (GIPLs), cruzipain, trans-sialidases (TS) and the mucin group TcSMUG L. The protection against the digestive enzymes may be performed by gp63 *via* its metalloprotease activity; TS may be involved in protection from glycolytic enzymes; and the mucin group TcSMUG S may perform roles of protease resistance.

Notably, most of the virulence factors molecules groups involved in the human immune system evasion are also responsible to assure parasite’s survival in their insect vector (**Figure 1**), despite being whole different hosts and environments. A few of the genes coding for some of these molecules, e.g., gp63, PPG and mucin may be traced back to precursor genes present in the non-parasitic near relative of trypanosomatids, *Bodo saltans*. Based on that fact, it is reasonable to speculate that the probable origin of parasitism and what would become the first systems of cell defense were originated by random genetic events of gene losses and gains in the trypanosomatid ancestor (Jackson et al., 2016). They allowed (1) the pre-existing molecules to change and adapt that protozoa to infect other hosts and (2) the creation/expansion of unique multigene families present in each parasite (Jackson et al., 2016). These events initially would allow the survival of these organisms inside insect’s intestines, followed by future adhesion roles that would permit them to attach, multiply and be transmitted to other hosts, establishing a monoxenous life cycle. Evidence of this gradual evolutionary step can be observed by the presence of the gp63 orthologs in monoxenous trypanosomatids and even in the free living kinetoplastid *B. saltans*, as previously mentioned in this review. Another example of that, could be the presence of precursor coding sequences in genomes of even more early monoxenous trypanosomatids, e.g., *Paratrypanosoma confusum* and *Leptomonas pyrrhocoris. P. confusum* was discovered infecting mosquitoes and is considered of great importance to the study of parasitism origins, due to its phylogenetic classification as an intermediate kinetoplastid, that lies between the free-living bodonids and parasitic trypanosomatids (Flegontov et al., 2013). Whereas *L. pyrrhocoris* is phylogenetically closer to *Leishmania* genus and insights from its genome may elucidate how *Leishmania* acquired its dixenous life cycle (Flegontov et al., 2016). Their annotated genomes [*Paratrypanosoma confusum* (Skalický et al., 2017) and *Leptomonas pyrrhocoris* (Flegontov et al., 2016)] available in the TriTryp database show coding sequences for LPG, gp63, PPG, cruzipain, TS and mucin, which are important virulence factors for both humans and insects, as mentioned previously in this review. Since these organisms only infect one host, the evolutionary event that led to the dixenous life cycle happened in separate occasions for the *Trypanosoma* and *Leishmania* genus (Jackson, 2015).

## Genetic Mechanisms Contribution to Gene Variability and Virulence in Trypanosomatids

Most of the proteins involved in the virulence in invertebrate and vertebrate hosts of *T. brucei, T. cruzi* and *Leishmania* spp., are part of multigene families generated by genome remodeling in these parasites. Among other roles during the infection, these genes are the basis for the specific cell surface content displayed by each parasite genus and may be associated with the disease in humans and survival inside insects (Jackson, 2015). This diversity is possible due to their genomic plasticity and evolutionary need to move through different hosts and be exposed to different environments during the life cycle. The evolutionary events that originated these genes could vary from a mutation by a single amino acid substitution to a whole chromosomal duplication (with their coding and non-coding regions), however, the gene duplication events are considered to be the main mechanism responsible for producing new material for evolutionary innovation (Andersson et al., 2015; Jackson, 2015).

These duplications can be achieved by different genomic processes. For instance, subtelomeric regions of *T. cruzi* are enriched with members of multigene families encoding (glyco)proteins (TS, RHS, MASP, mucins, DGF-1 and GP63) that are subjected to intense genomic rearrangements, typical for that chromosomal area (Kim et al., 2005; Moraes Barros et al., 2012). Moreover, other non-syntenic regions (disruptive compartments) in *T. cruzi* genome, composed of TS, MASP and mucins multigene families, have also a role in the homologous recombination, serving as an homology site (Berná et al., 2018). These regions are susceptible to double-strand breaks of DNA, by retrotransposon nucleases, that tend to be repaired by homologous recombination. However, if during the repair, non-homologous chromatids are used, new gene variations may be created (Herreros-Cabello et al., 2020). If these variants are proved to be functional, it can promote new host-parasite interactions that would lead to parasite evasion in their hosts (Herreros-Cabello et al., 2020). It is suggested that part of gene expansion of virulence factors comprised of multigene families located in the subtelomeric regions, like VSG in *T. brucei* (El-Sayed et al., 2000), trans-sialidases (Freitas et al., 2011), mucins and MASPs (Mucin-Associated Surface Proteins) in *T. cruzi*, are due to their chromosome location (Herreros-Cabello et al., 2020).

Another process that can also increase gene diversity is the gene conversion mechanism that promotes the duplication of genes in tandem arrays from one common gene, but with sequence divergence (Jackson, 2007). Regardless of the gene duplication mechanism that generates the paralogs, these events are responsible to produce the materials for the evolutionary improvement that allows organisms to adapt and increase in complexity (Andersson et al., 2015; Jackson, 2015). Following the gene duplication event, the new copy may (1) present a novel function, (2) lose the function due to a mutation and be silenced as a pseudogene or (3) keep the function of the original gene. However, the latter case may be accompanied by accumulative mutations that would reduce their total capacity to the level of the single-copy ancestral gene (Lynch and Conery, 2000).

The presence of this genomic duplication is clearly seen in the abundant tandem arrays in trypanosomatids, especially in proteins involved with virulence, such as VSG in *T. brucei* (Jackson, 2007), gp63 in *Leishmania* spp. (Castro Neto et al., 2019) and mucins (Buscaglia et al., 2006) and TS in *T. cruzi* (Kim et al., 2005; Moraes Barros et al., 2012; Berná et al., 2018). These events are highly common in trypanosomatids, probably as a mechanism of increasing polycistronic transcription abundance for genes which are in need for a high expression rate (Jackson, 2007). These processes that lead to the genetic diversity of these parasites proteins may directly affect the outcome of host-parasite interactions, since they may produce different strains, which cause diverse responses in their hosts (Ponte-Sucre, 2016).

### Genetic Events Associated With VSG Variation in *T. brucei*

For *T. brucei*, the high genetic variation of its main antigenic protein (VSG) is a result of homologous recombination or transcriptional switching among approximately 15 different VSG expression sites with a repertoire of approximately 1,000-2,000 different genes. They possess long transcription units with other expression site-associated genes (ESAGs) apart from VSG, which also plays a role in *T. brucei* antigenic variation (Reis-Cunha et al., 2017; Sima et al., 2019). To do that, silent VSG genes are copied and placed into expression sites, replacing the old gene (Rudenko, 1999). This event creates several categories of VSG genes, which are composed in its majority by pseudogenes, that are used by the parasite to produce chimeric genes by gene conversion and increase VSG variability (Roth et al., 1989). Despite the gene conversion method, silent VSG genes may also switch, through telomere exchange, from a silent to an active VSG expression site (Rudenko, 1999), by a DNA repair process (Horn, 2014; Sima et al., 2019).

### Impact of Genetic Polymorphisms in Subsets of Proteins From *T. cruzi* Multigene Families Involved in the Parasite Virulence

The trypanosomatids (*T. brucei* and *Leishmania* spp.) have most of their virulence surface proteins as part of multigene families. In *T. cruzi* the main representatives of this group are the TS and the mucins. The TS and TS-like genes comprise a number around 1,500 genes depending on the strain, that can be separated by sequence similarity into 8 groups (Freitas et al., 2011). This provided the parasite an array of different functions which are present in many different stages of the life cycle (Herreros-Cabello et al., 2020).

Relative to the mucins, despite having groups of genes being differentially expressed in the insect and mammalian hosts, they tend to present wide sequence polymorphisms (Buscaglia et al., 2006). These variations are more common in mucins expressed in the mammalian host than in the insect host, which may be attributed to their localization by the subtelomeric regions of the chromosome and immune system pressure during infections. Besides being used to divert recognition by the immune system, these variations also generated differences in the genome to the point to differentiate these proteins in distinctive function groups (De Pablos and Osuna, 2012). The genes expressed in mammals also are divided into two groups: TcMUC I (predominant in amastigotes) and TcMUC II (present in trypomastigotes). In their genomic structure they possess a central region with high variability sequence, which is long in TcMUC II and short TcMUC I. This region is known for having binding sites for glucose and sialic acid and may be responsible for the high glycosylation rates of these proteins, when compared to the ones expressed in the epimastigote stage (De Pablos and Osuna, 2012; Herreros-Cabello et al., 2020).

Another group of proteins that also have a hypervariable central region in their sequence are the MASPs. In terms of gene numbers, they are considered the second largest protein family in *T. cruzi*. Despite having a highly conserved N- and C- terminal regions, they are cleaved during the protein maturation, leaving only the hypervariable region exposed at the parasites’ membrane (El-Sayed, 2005; Bartholomeu et al., 2009). This case of high variation can also be attributed to the subtelomeric regions of the genes, but also to probable cases of homologous recombination due to the highly conserved UTR sequences (Souza et al., 2007).

### Surface Proteins Variability and Their Role in *Leishmania* spp. Virulence

*Leishmania* does not possess the same expression mechanisms as the VSGs of *T. brucei*. They rely in other genetic events, like gene sequence variation and expansion throughout the genome, to contribute to host immune evasion. The carbohydrate moieties of molecules like PPGs (McConville et al., 2002) and LPGs, for instance, present high variation among themselves, which increases their chances of interacting with a myriad of components in the insect and vertebrate hosts. These variations in LPGs are so remarkable that they can be stage and species specific, besides presenting different functionalities (Franco et al., 2012; Bifeld and Clos, 2015; Valente et al., 2019). Studies in *L. major* metacyclic form show that this stage is resistant to complement mediated lysis, whereas the promastigote form present in the insect is highly susceptible. This difference is conferred by changes in the molecule PG moiety that is approximately twice as long in the metacyclic form, when compared to the ones present on the promastigote. Despite this modification impairs the promastigote survival of the complement attack, it is what allows the parasite to detach from the insect gut prior transmission and migrate to their mouthparts (Franco et al., 2012; Bifeld and Clos, 2015). In addition, variations to the LPG side chains carbohydrates have also been implicated in species specific parasite-vector attachment. It has been shown that the vector *Phlebotomus papatasi* ability to only transmit *L. major* is linked to the parasite’s specific LPG structure, which comprises multiple terminally exposed β-linked galactose residues, responsible for binding to the vector’s gut. *In vitro* assays with parasites that do not possess this carbohydrate side chain patterns have failed to bind to *P. papatasi* mid guts (Sacks, 2001).

The same principle for LPGs can be applied for gp63 proteins, which are expressed by a variety of species-specific copy number and coding sequences, as shown in **Table 2**. Notably, there is a substantial increase in the number of coding sequences for these genes in *L. braziliensis*, which is a species belonging to subgenus *Viannia*, when compared to species of the *Leishmania* subgenus (*L. major, L. infantum and L. mexicana*), as observed in TritrypDB database and early genomic studies (Victoir et al., 1995; Victoir et al., 2005).

**Table 2:** Gp63 gene repertoire among the main medical interest species of *Leishmania* distributed by subgenus and chromosome.

Species	Subgenus	Chromosome 10 genes	Chromosome 28 genes	Chromosome 30/31 genes	Total genes
*L. major*	*Leishmania*	4	1	1	6
*L. infantum*	*Leishmania*	13	2	1	16
*L. mexicana*	*Leishmania*	5	1	1	7
*L. braziliensis*	*Viannia*	33	–	6	39

These genes are organized in tandem and are generally distributed in three chromosomes (10, 28 and 30/31), depending on the species. Most of the genes are located on chromosome 10, as shown in **Table 2**, varying in number from 4 to 33 and with fewer genes (1 or 2) on chromosomes 28 and 30/31. A phylogenetic study showed that the genes present on chromosomes 28, 30 and 31 are more similar to genes present on monoxenous trypanosomatids, like *Leptomonas* and *Chrithidia* (Castro Neto et al., 2019). This may imply that these genes are probably the ones with a functional role during the invertebrate host stage of the life cycle, but more studies are needed to confirm this hypothesis.

Overall, these genes seem to have expanded forming paralogs by recombination events mainly on chromosome 10 of *L. braziliensis*, which is proposed to be more involved with the mammal host infection (Castro Neto et al., 2019). These events, besides increasing the numbers, produce sequence variation that may also contribute to parasite’s virulence. Despite maintaining a conserved core domain, studies performed in *L. braziliensis* showed that regions encoding surface peptides possibly involved in host–parasite interaction, presented high variability (Victoir et al., 2005; Castro Neto et al., 2019). However, due to current sequencing methods and the high similarity rate among these genes, more studies are needed to point out the actual number, gene variation and functional role of these different gp63 variants in their hosts. We speculate that this would be an adaptive strategy of the parasite to improve virulence during the mammal host infection and also allow them to infect a myriad of different insect vectors.

## Concluding Remarks

The proteins directly or indirectly involved in host parasite mechanisms and immune system evasion today, in invertebrate and vertebrate hosts, may have been originated by genetic events that made this possible during millennia of evolution. We can speculate that these variations, mainly on surface proteins involved with host-parasite interaction traced back to early kinetoplastids, allowed the once free-living protozoans to start infecting insects producing new species, that later could infect vertebrate hosts. This was probably made possible due to the genomic events described on this review (gene conversion, reciprocal recombination events and mutations, associated with duplications that led to the creation of multigene families that proved to be essential during the parasite’s evolution). These non-stopping genetic variations are constantly producing gene variants that may improve the host-parasite interaction in their hosts, facilitating parasite survival and proliferation.

There is no question on the importance of virulence factors studies for the host parasite relationship in vertebrate hosts. However, the roles of these molecules in the insect vector requires further studies, to better characterize them or find novel factors involved in this stage of the life cycle. Understanding the genomic events that led to their variations and allowed them to have groups of molecules with specific roles on different hosts may contribute to the analyses of these proteins’ functions and features, which may open new possibilities to the development of new translational studies relative to the control of insect vectors in endemic areas. In addition, these comprehension of genetic mechanisms or events that contributed to the virulence of these parasites can give an important contribution to the improvement in the search of novel targets in drug, vaccine, and diagnostic research. It is important to not label these groups as a whole and be aware of their sequence variation and subsets of molecules with actual and specific roles in their respective stage in the parasite’s life cycle.

## Author Contributions

ACN wrote the manuscript and prepared the table and figure. JS and RM reviewed and edited the manuscript, table, and figure. All authors contributed to the article and approved the submitted version.

## Acknowledgments

We would like to thank Fundação de Amparo a Pesquisa do Estado de São Paulo (FAPESP) (Grant: 2016/15000-4 and fellowship: 2019/23302-9) for their financial support. The figure was created with BioRender.com.

## Conflict of Interest

The authors declare that the research was conducted in the absence of any commercial or financial relationships that could be construed as a potential conflict of interest.

## Publisher’s Note

All claims expressed in this article are solely those of the authors and do not necessarily represent those of their affiliated organizations, or those of the publisher, the editors and the reviewers. Any product that may be evaluated in this article, or claim that may be made by its manufacturer, is not guaranteed or endorsed by the publisher.
